# So Happy Together: A Review of the Literature on the Determinants of Effectiveness of Purpose-Oriented Networks in Health Care

**DOI:** 10.1177/10775587221118156

**Published:** 2022-08-24

**Authors:** Robin Peeters, Daan Westra, Arno J. A. van Raak, Dirk Ruwaard

**Affiliations:** 1 Maastricht University, The Netherlands

**Keywords:** networks, effectiveness, health care, review, interorganizational

## Abstract

While purpose-oriented networks are widely recognized as organizational forms to address wicked problems in health care such as increasing demands and expenditure, the associated literature is fragmented. We therefore reviewed empirical studies to identify the determinants of the effectiveness of these networks. Our search yielded 3,657 unique articles, of which 19 met our eligibility criteria. After backward snowballing and expert consultation, 33 articles were included. Results reveal no less than 283 determinants of effective health care networks. The majority of these determinants are processual and involving professionals from the operational level is particularly salient. In addition, most studies relate determinants to process outcomes (e.g., improved collaboration or sustainability of the network) and only a few to members’ perception of whether the network attains its goals. We urge future research to adopt configurational approaches to identify which sets of determinants are associated with networks’ ability to attain their goal of addressing wicked problems.

## Introduction

The “wicked problems” that health care systems face, such as increasing expenditures, a lack of high-value service delivery, and increasing demands challenge their sustainability (Organisation for Economic Co-operation and Development [OECD]/EU, 2018; Provan & Lemaire, 2012). Each of these wicked problems constitutes challenges that no single organization can manage on its own but requires the collective action of multiple stakeholders (Provan & Milward, 2001). Since their introduction as a form of organizing between hierarchies and markets (Powell, 1990), networks have been described as an appropriate way of organizing to address such problems (Westra et al., 2017). For example, delivering value to patients requires health care organizations to collaborate across different sectors to lower costs and improve outcomes (Porter, 2010). Networks thus play a crucial role in the sustainability of health care systems. Policy spurs to integrate care delivery (Burns et al., 2022) have caused networks to become increasingly prominent in health care (Young, 2012). Given the importance attached to these networks to solve health care’s wicked problems and the resources invested in them, it is imperative to understand how to organize them in a way that maximizes their effectiveness.

Over the past decades, networks have been widely studied (Borgatti & Halgin, 2011). Examples in health care include multi-stakeholder alliances (e.g., D’Aunno et al., 2017) cross-sectoral partnerships (Brewster et al., 2018), patient referral networks (e.g., Mascia et al., 2015), and collaboration between care professionals (e.g., Tasselli, 2014). Since the seminal paper of Provan and Milward (1995) on network effectiveness in mental health systems, scholars have theorized that network effectiveness can be operationalized at the community, network, or participant level (Provan & Milward, 2001) and in terms of processes or outcomes (Head, 2008). Empirical and conceptual work in management and public administration literature furthermore suggests that many determinants can influence network effectiveness, including process determinants (e.g., trust), structural determinants (e.g., ties between actors), and contextual determinants (e.g., the institutional environment; Nowell & Kenis, 2019; Turrini et al., 2010). Several of these determinants have been studied empirically in health care (e.g., Alexander et al., 2003; Bainbridge et al., 2011; Hearld & Alexander, 2014), but this literature is fragmented due to the many different labels and definitions. An overview of the determinants of network effectiveness will increase our understanding of how organizations in health care can collaborate in networks to address wicked problems and identify avenues for future research.

We aim to assess the current state of the literature and identify the determinants for the effectiveness of deliberately formed networks addressing wicked problems in health care. Therefore, this study addresses the following research question:

**Research Question 1:** What are the determinants of the effectiveness of health care delivery networks?

By answering this question, we seek to contribute to the broader scientific understanding of networks in health care as well as to the way health services are organized in practice. Scientifically, our study can form the foundation for a research agenda around fostering successful networks in health care. As organizations increasingly collaborate in networks to increase the value of health services, our findings can furthermore serve as a guide for health care organizations and their managers to deliberately organize networks in a way that can maximize their effectiveness.

## New Contribution

With this study, we make two contributions to the literature on, and applications of, purpose-oriented networks in health care. First, we provide a comprehensive overview of the current state of our knowledge on the determinants of the effectiveness of purpose-oriented networks in health care. That is, we identify the determinants that have been associated with effective networks in existing research. Such overviews have been instrumental in identifying knowledge gaps and directions for future research, for example, in management and public administration literature (Ansell & Gash, 2007; Bryson et al., 2015; Parent & Harvey, 2009; Planko et al., 2017; Popp et al., 2014; Turrini et al., 2010). By focusing specifically on networks in the health care sector, we seek to synthesize the current knowledge on the determinants of purpose-oriented networks in the health care sector in which networks are increasingly established (Young, 2012) and play an important role to increase the value of care delivery (Porter, 2010). In doing so, we are furthermore able to identify directions for future research in the sector. Second, we explore how these determinants are linked to various types and levels of effectiveness. By identifying the measure of effectiveness in empirical work, our study shows that the determinants identified in the literature help us to understand how to collaborate well, not how networks can attain their goals to address wicked problems. This finding suggests that both researchers studying health care networks as well as managers and professionals participating in these networks should reflect on how to ensure that they successfully reach their goals.

## Theoretical Framework

Throughout the literature, different labels are used interchangeably to define networks and for many labels, multiple definitions exist (Lemaire et al., 2019). Recently, Nowell and Milward (2022) developed a taxonomy of networks, distinguishing three classes of networks: structural-oriented networks, system-oriented networks, and purpose-oriented networks. Structural-oriented networks represent emergent social structures, have no set boundaries, and do not exist other than the consequences they have for the actors in the network itself (Nowell & Milward, 2022). The relationships between actors can be mapped using Social Network Analysis and are also present in system-oriented and purpose-oriented networks (Nowell & Milward, 2022). In health care, for example, care professionals such as doctors and nurses have informal relationships on a personal level in social networks that can shape professionals’ work satisfaction, knowledge transfer, or diffusion of innovations (Tasselli, 2014). System-oriented networks represent the actors and their interactions associated with (but independent from) a (policy) issue. The actors do not have a collective identity but the network does represent a system that is associated with an outcome (Nowell & Milward, 2022). In health care, efforts to increase integration in existing health systems include, for example, the seminal work of Provan and Milward (1995) regarding mental health systems and Brewster et al. (2019) on elderly care systems. In addition, in patient referral networks, patients are transferred from one hospital to another with the aims of increasing the quality of care or gaining strategic advantages (Mascia et al., 2015).

Purpose-oriented networks (Carboni et al., 2019) are deliberately formed, self-actualized entities, meaning the network is self-referencing (using an official name and artifacts such as a website) and members associate with the network (Nowell & Milward, 2022). These networks have bounded membership which exists because members share a common goal, and therefore, the network has clear boundaries (Nowell & Milward, 2022). These networks typically involve a range of stakeholders such as care provider organizations, purchasers, government agencies, and (representatives of) consumers or client groups (e.g., Alexander et al., 2016) that pursue a common goal they cannot solve on their own. These goals are often related to increasing the value of health care (i.e., higher quality per cost; Porter, 2010) or enhancing the Triple Aim (i.e., improving the patient experience of care and the health of populations and reducing per capita costs; Berwick et al., 2008). Examples include multi-stakeholder alliances (D’Aunno et al., 2017), health care delivery networks (Nicaise et al., 2021), or community health partnerships (Alexander et al., 2003). Henceforth, we consider purpose-oriented networks a collection of three or more autonomous organizations working together deliberately and specifically to achieve a common goal, which they expect to reach more easily if they collaborate (Nowell & Kenis, 2019; Provan et al., 2007).

The effectiveness of networks can be evaluated at multiple levels, including the level of the community, the whole network, and the organization and participant (including clients; Provan & Milward, 2001). However, it can have a different meaning at each level. At the community level, for example, the effectiveness of networks in health care may be evaluated according to Triple Aim outcomes (Berwick et al., 2008), while at the organizational/participant level, it may entail improving the value of care delivery (i.e., lowering cost and improving quality of care services; Porter, 2010). At the network level, it can be evaluated according to the range of care services provided or the cost of maintaining the network (Provan & Milward, 2001). Purpose-oriented networks can furthermore be evaluated along two dimensions (Head, 2008). The first dimension involves the generated outcomes such as the degree of goal attainment (e.g., Turrini et al., 2010), which can be measured at all three levels. The second dimension involves the processes within the network, such as decision-making processes and the ability to align and manage different perspectives (Head, 2008; Turrini et al., 2010), which are typically measured at the level of the network itself. The comprehensiveness of the concept makes it difficult to operationalize and measure network effectiveness in practice (Smith, 2020). This is illustrated by the fact that empirical research typically measures effectiveness at one of these levels, although the levels are interrelated and effectiveness may be generated at one level at the expense of effectiveness at another level (Head, 2008; Smith, 2020), meaning for example that effectiveness at the level of the network can be created at the expense of patient-level outcomes.

Various scholars have also researched determinants of networks and their effectiveness. Kenis and Raab (2020) identified six frameworks in management and public administration literature that review the determinants of network effectiveness (Ansell & Gash, 2007; Bryson et al., 2015; Parent & Harvey, 2009; Planko et al., 2017; Popp et al., 2014; Turrini et al., 2010). More recently, Smith (2020) synthesized the theoretical reflections of network effectiveness and its determinants in an additional framework. These frameworks show three common groups of determinants, including the network’s processes, context, and structure (see also Nowell & Kenis, 2019; Turrini et al., 2010). The first group of determinants of network effectiveness, the processes, constitutes the largest group of determinants and relates to the collaborative processes occurring inside the boundaries of the network itself. Examples include trust, commitment, a shared understanding of the problem and goal, communication mechanisms, and leadership (e.g., Ansell & Gash, 2007). The second group, the context, relates to environmental and historic dimensions that occur outside the boundaries of the network itself and includes determinants such as the (institutional) environment (e.g., Parent & Harvey, 2009), pre-existing relationships (e.g., Turrini et al., 2010), and system stability (e.g., Ansell & Gash, 2007). Third, the group structure defines the boundaries of the network and the formal design of the network and includes determinants such as the governance structure (e.g., Bryson et al., 2015) and the composition of the network members (e.g., Planko et al., 2017).

## Method

Our study aims to identify the characteristics that influence network effectiveness and identify current gaps in the literature on this topic. Because scoping reviews are especially suitable for these purposes (Munn et al., 2018), we conducted a scoping review following the Joanna Briggs Institute’s scoping review guidelines (Peters et al., 2020).

### Search Strategy

We searched the PubMed, Cumulative Index to Nursing and Allied Health Literature (CINAHL), and PsycInfo databases in October 2020 and repeated our search in February 2022. The search strategy was initially constructed and performed in PubMed in consultation with an information specialist from the lead author’s university library. The various labels used to describe the different types of networks (structural-oriented, system-oriented, and purpose-oriented; Lemaire et al., 2019) in existing literature generate an unwieldable number of possible free search terms and combinations. Therefore, we limited our searches to Medical Subject Heading (MeSH) Terms. We identified these MeSH Terms by exploring the MeSH Tree, selecting potentially relevant MeSH Terms, and assessing whether the results of each MeSH Term yielded relevant results. In PubMed, the search included the following Medical Subject Heading (MeSH) terms: Community Networks, Intersectoral Collaboration, Health Care Coalitions, Interinstitutional Relations, and Multi-Institutional Systems. We took the same approach to identify the relevant Exact Major Subject Headings (EMSH) in CINAHL (Interinstitutional Relations, Collaboration, Shared Governance, Multi-institutional Systems, and Coalition) and PsycInfo (Collaboration, Cooperation, Coalition Formation, and Integrated Services).

To further narrow the search, we added three parts to the search string that remained the same in all three databases. First, we added two free terms in the title and abstract (i.e., organization* OR organisation*) and a MeSH or EMSH term (i.e., Organizations) to include articles studying interorganizational as opposed to interpersonal networks. Second, we added the free terms “health care,” “health,” and “care” to exclude studies in other empirical settings. Finally, we added the terms effectiv*, succes*, evaluat*, consequen*, impact*, and perform* in the title or abstract to include articles that studied the effectiveness of networks. We searched for articles published since 1995 (following Provan & Milward, 1995) and in English. In total, our database search identified 3,936 articles of which 279 were removed due to deduplication, leaving 3,657 articles for eligibility assessment.

We assessed the eligibility of articles in two rounds. First, we screened the title, keywords, and abstract. Studies were excluded if they (a) did not investigate a network of any type; (b) did not investigate an interorganizational network but, for example, an interpersonal or interstate network; (c) did not investigate a purpose-oriented network but a structural-oriented or system-oriented network; (d) investigated a network of which the goal was not primarily concerned with improving the value (i.e., improving outcomes or reducing costs) of health care delivery; (e) did not investigate determinants influencing network effectiveness, meaning articles reporting solely if networks were effective or not without reporting the determinants in their results were also excluded from the review; (f) were not peer-reviewed, published articles but, for example, an editorial or a book; (g) were not empirical studies; (h) did not have a clearly defined methodological approach; or (i) had no available full-text in English.

While we applied the taxonomy of Nowell and Milward (2022) to identify articles studying purpose-oriented networks, these groups are analytical and different scholars thus might have different interpretations of how to apply these characteristics to empirical phenomena. Therefore, all four authors had regular discussions on the application of these criteria. To ensure exclusion criteria were applied consistently, two researchers independently assessed the title, keywords, and abstract of 100 randomly chosen articles (Waffenschmidt et al., 2019). The interrater reliability was 85% (85/100). Any disagreements were discussed until consensus was reached and the exclusion criteria were refined where necessary. One researcher applied these criteria to the remaining articles. Second, we assessed the eligibility on full-text using the same exclusion criteria. The two researchers assessed 22 randomly chosen articles on full-text to ensure that the criteria were applied consistently, which resulted in interrater reliability of 86% (19/22). One researcher assessed the remaining articles. Additional uncertainties were discussed with the second researcher and subsequently with all authors. In total, 3,657 articles were screened on title and abstract, of which 3,481 were excluded. After a full-text eligibility assessment (*n* = 176), 157 articles were excluded resulting in 19 articles that met our eligibility criteria.

**Figure 1.**
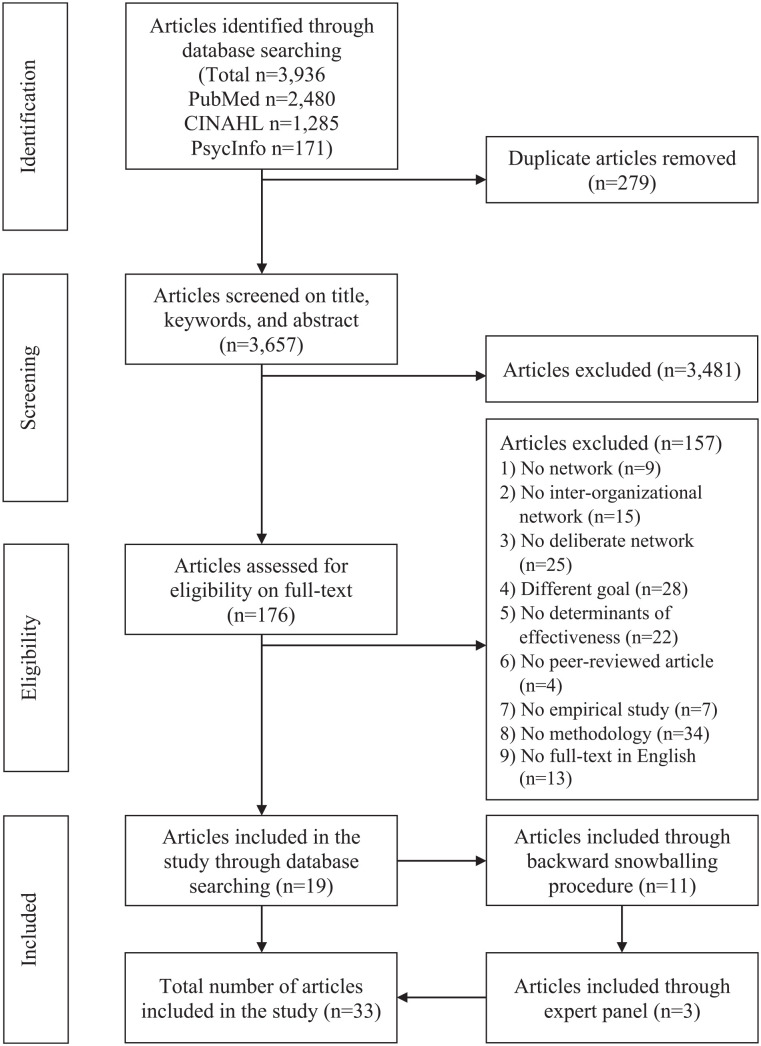
PRISMA Flow Diagram of the Review Process.

To identify additional articles that were not captured using the database search, we took two additional steps. First, we performed a backward snowballing procedure (Wohlin, 2014) using the reference lists of the included articles to identify additional articles that met our eligibility criteria. This resulted in the inclusion of an additional 11 articles. Second, we constructed a list of 19 experts from different countries known to have published on interorganizational, purpose-oriented networks. We contacted the experts via e-mail for their input on the list of included articles so far and for additional articles that might meet the eligibility criteria to ensure no key articles were missing. Thirteen experts responded. We assessed all suggested articles using the eligibility criteria. Moreover, we performed an additional forward snowballing procedure (i.e., a cited references search, see Wohlin, 2014) of Provan and Kenis (2008) in Web of Science. The expert input led to the inclusion of an additional three articles, resulting in a final total of 33 included articles in the study. Articles identified using the snowball procedure and expert consultation were not captured by our database search mostly because they did not use any MeSH Terms or because of the organization* string. An overview of the full identification and selection process is visualized in the PRISMA flow diagram in Figure 1. An overview of the exclusion reasons for all articles and our data extraction can be found here (Peeters et al., 2022).

### Data Extraction and Analysis

Following the Joanna Briggs Institute’s scoping review guidelines (Peters et al., 2020), we extracted the year of publication, journal, country of origin, theoretical concepts, methodology, intervention details, outcome details, and the key findings. For the intervention details, we extracted the characteristics of the studied networks. That is, we extracted the number of networks included, the goal of these networks, the number of members in the network, and the type of members. For the outcome details, we extracted the operationalization and measurement of effectiveness and the degree to which the studied networks were effective. Finally, we extracted the determinants of network effectiveness that were reported and categorized them as facilitating or inhibiting according to the study’s findings. One researcher coded the data on the operationalization of effectiveness into the groups’ outcomes measure, process measure, goal attainment, sustainability of the network, or others. The same researcher coded the data on the determinants of network effectiveness into common themes and, subsequently, into the processes, context, or structure group. The coding was first discussed with a second researcher and eventually with all authors and adapted where necessary. Determinants that fit into more than one group were assigned to a single group based on consensus between the authors.

## Results

Table 1 presents an overview of the study characteristics of the included studies. All studies except one (Hearld et al., 2013), published in Nonprofit Management and Leadership were published in health or health care-specific journals. The majority of studies were conducted in the United States (*n* = 20), eleven in Europe, one in Australia, and one study was conducted across different locations worldwide (see Table 1). The studies had sample sizes at the network level ranging from one to 25.

**Table 1.** Study Characteristics.

**#**	Authors and year	Journal	Country of origin	*N* (network)
1	Alexander et al. (2010)	*Health Education & Behavior*	United States	4
2	Alexander et al. (2016)	*The American Journal of Managed Care*	United States	16
3	Alexander et al. (2003)	*Health Care Management Review*	United States	4
4	Bainbridge et al. (2011)	*Journal of Palliative Care*	Canada	1
5	Bazzoli et al. (2003)	*Medical Care Research and Review*	United States	25
6	Bunger & Gillespie (2014)	*Health Care Management Review*	United States	1
7	Conrad et al. (2003)	*Medical Care Research and Review*	United States	25
8	D’Aunno et al. (2017)	*Health Care Management Review*	United States	16
9	D’Aunno et al. (2019)	*Health Care Management Review*	United States	15
10	Guihan et al. (1995)	*Medical Care*	United States	20
11	Harlock et al. (2020)	*Journal of Health Services Research & Policy*	United Kingdom	16
12	Hasnain-Wynia et al. (2003)	*Medical Care Research and Review*	United States	25
13	Hearld & Alexander (2014)	*American Journal of Community Psychology*	United States	17
14	Hearld & Alexander (2020)	*Health Care Management Review*	United States	15
15	Hearld, Alexander, Beich, et al. (2012)	*American Journal of Managed Care*	United States	14
16	Hearld et al. (2013)	*Nonprofit Management and Leadership*	United States	14
17	Hearld, Alexander, & Mittler (2012)	*Health Care Management Review*	United States	14
18	Hearld et al. (2015)	*Health Care Management Review*	United States	16
19	Hearld et al. (2018)	*Journal of Health Organization and Management*	United States	8
20	Hearld et al. (2019)	*Journal of Health Organization and Management*	United States	8
21	Hermans et al. (2019)	*International Journal of Integrated Care*	Belgium	15
22	Kristensen et al. (2019)	*Nordic Journal of Psychiatry*	Denmark	14
23	Lemieux-Charles et al. (2005)	*The Gerontologist*	Canada	4
24	Ling et al. (2012)	*International Journal of Integrated Care*	United Kingdom	16
25	Lorant et al. (2017)	*Administration and Policy in Mental Health and Mental Health Services Research*	Belgium	19
26	Metzger et al. (2005)	*Health Education & Behavior*	United States	25
27	Nicaise et al. (2021)	*Journal of Interprofessional Care*	Belgium	19
28	Nikbakht-van de Sande et al. (2005)	*Journal of Palliative Medicine*	The Netherlands	8
29	Sharek et al. (2007)	*Joint Commission Journal on Quality and Patient Safety*	United States	20
30	van Eyk & Baum (2002)	*Health and Social Care in the Community*	Australia	1
31	van Raak et al. (2008)	*Health & Place*	The Netherlands	1
32	Willem & Gemmel (2013)	*BMC Health Services Research*	Belgium	22
33	Wilson et al. (2003)	*Joint Commission Journal on Quality and Patient Safety*	Australia, France, the Netherlands, Norway, Sweden, United Kingdom, and United States	Not specified

### Determinants Related to Network Effectiveness

Table 2 provides an overview of the determinants of network effectiveness according to the studies in our review, categorized according to the groups processes, context, and structure, and whether they inhibit or facilitate network effectiveness. In total, we extracted 283 determinants from the included articles, clustered into 30 themes. While some determinants might be considered into multiple groups or might overlap, we choose the group most suitable for each determinant. For example, we found both the availability of human resources, such as care professionals, which we classified as context because they are resources that are often scarcely available to the network and organizational members of the network which we classified as a structure because they define the composition and thus the boundaries of the network. In what follows, we elaborate on the themes that are most often mentioned in each group.

**Table 2:** Summary of Determinants of Network Effectiveness.

Themes	Inhibiting determinants (article number)	Facilitating determinants (article number)
Processes (total 185)		
Problem, goal, and vision (19 articles / 30 determinants)	No clear/shared goal, vision, or expectations (2, 11, 21, 29) Too many or complex initiatives (2, 5, 24)	Consensus on and shared understanding of the goal, vision, and expectations (2, 3, 9, 11, 13, 24, 26, 27, 28, 30) Congruence between network and member goals (2, 18) Creating and clarifying value for members (2, 3, 19, 24) Attention to long-term directions (3) and reconciling short- and long-term objectives (1) Clearly focused intervention and well-defined target population (7) Taking an active approach in communicating with stakeholders to educate them about the alliance, its goals, strategies, and how these activities would affect the stakeholders (18) Developing products and initiatives together (17) A topic consistent with local or national priorities, fitting the specific setting (33) Including experts in generating ideas while also allowing members to generate their own ideas (33)
Leadership (16/23)	Turnover in leaders of the alliance or in member organizations (5, 18) Leaders do not have the required leadership skills: keeping the group focused on the objectives (12); managing interactions, energizing members, sustaining enthusiasm of members (29) Key leaders not involved in network decision-making or unaware of its initiatives (2)	Leadership that is effective (8, 12, 18), distributed (3), continuous (3), action-oriented (5), competent (7), committed (7), strong (11), ethical (12), empowering (13), stable (15), supportive (23), clinical (24), and present (28) Engaging leaders from a broad and representative group of committed individuals and, at the same time, direct the alliance in an effective fashion toward its ultimate objectives (1) Changes in program manager (18) Leading through vision (26)
Planning/ coordination (13/18)	Time scales that are too short (24) or do not match member time frames (29) Poor task coordination (5) Higher rating of the effectiveness of communication and coordination mechanisms relative to other members (10)	Defining roles and responsibilities (1, 5, 11) Generation of momentum from initial successes (3) Task-oriented focus (3) Developing standards of practice (4) Higher number of action steps in an initiative (5) Strong integration and linkage of intervention components or activities with each other and with the broader goals (3, 7) Broad and centralized information sharing (23) Planning specific improvement routes (28) Arranging case discussions (28) Involving all organizational members as early as possible in activities (30) Having strong project management and support driving teams (33)
Decision-making (12/17)	No integrated management for inter-sectoral decision-making (22)	Collaborative decision-making including all members (8, 11, 13, 16, 17, 26, 30) Open and transparent decision-making (16, 17, 26) Establishing formal decision-making frameworks (16, 32) Good and helpful decision-making structure (12, 15) Congruence between types of decisions and decision-making processes (16) Satisfaction about speed of decision-making (28)
Operational level (10/14)	Health care professionals reluctant to engage (24) Health care professional’s interest waned due to loss of momentum (29)	Formally involving health care professionals (21, 23, 24, 28, 33) Creating awareness and shared beliefs about the (personal) benefits among the operational level (11, 17, 24) Educational opportunities for professionals (4, 24) Encouraging personal contact/connecting healthcare workers (21) Staff receiving support from the agency’s management (30)
Commitment (10/13)	Network participation is seen as obligation (2) Inconsistent attendance at network meetings (23) Competing priorities (29) Lack of willingness to be proactive (29) Interest waned due to loss of momentum (29) Lack of incentives to encourage cooperation (29)	High levels of participation/involvement in network activities (13, 17, 18) Organizational commitment or institutional ownership (3, 4) Highly motivated partners (7) Willingness to share information (11)
Trust (8/8)	Lack of trust (21, 29)	High levels of trust between members (6, 7, 11, 15, 20, 26)
Assessments (7/10)	Lack of defined measures of success or plan to measure success (29) Lack of historical data for process validation and making assumptions with little ability to confirm them (29) Change in measurement tool (29)	Common assessment and/or reporting tools (4, 22, 33) Documentation of value created and value added (3) Explicit, ongoing outcome measurement (7) Including dissemination targets in program development (20) Collect data and perform audit work before the start of the collaboration to develop starting points and a baseline (33)
Capacity and competencies (7/8)	Lack of staff and capacity (11) Lack of knowledge on each other’s area of work and competence (22) Lack of experience in multidisciplinary work (30)	Experience with collaboration (10) Aligning internal competencies with context (19) Balancing local capacities with system-wide goals (20) Competence development of employees also on inter-sectoral collaboration (22) Participants feel capable of making a favorable contribution to the network (28)
Institutional logics/ common culture (6/8)	Differences in cultures, values, opinions, procedures, and politics (11, 22, 29, 30) Changes in perceived professional boundaries/roles (24) “Them and us” attitudes (30)	Shared values and common culture (22, 24) Investing in bringing everyone up to speed and channeling competing perspectives (1) Staff feeling permitted to take risks (24)
Conflict/ tension (resolution) (6/6)	Less agreement/disagreement between members (10, 12) Difficulty maintaining healthy relationships between members (29)	Using conflict avoidance instead of conflict resolution (5) Avoidance of blame (11) Management of conflicts (13)
Communication mechanisms (5/8)	Higher rating of communication effectiveness relative to other members (10) Lack of personal contact among healthcare workers from different organizations (21) Lack of formal mechanism with regard to the exchange of information (21)	Communication of value added (e.g. through business case of media) (3) Establishing a climate where (divergent) ideas can be freely exchanged (16) Clear, consistent communication (24) Efforts to engage all participants (24) Regular updates on progress (24)
Authoritative texts (5/6)	Lack of agreements and guidelines for collaboration (21) Lack of a business case (20)	Putting agreements on record (28, 30) Signing a voluntary agreement (4) Presence of policy documents (28)
Technology (5/5)	Lack of shared information systems or common platforms (22, 29) Conflicting IT systems between members (24)	Shared information systems/data linkage (4, 24)
Organizational learning (4/6)	Resistance to change (7, 29) Divergence between existing and new routines (31)	Convergence between existing and new routines (31) Having members share improvement strategies (33) Adapting learning activities to organizational needs/preferences (33)
Internal legitimacy (4/5)	Differences in member perceptions of the network’s impact (10) Low levels of internal and external legitimacy (32)	Perceived fairness in the distribution of benefits and costs (16,17) Begin building fairness early (16)
External legitimacy (4/4)	Inability to develop a clear community identity (2) Low levels of internal and external legitimacy (32)	Network promotion and communication (4) Credibility of the alliance, the constituent organizations, and the alliance leaders (15)
Power (im)balances (3/4)	Members perceiving to have lower influence compared to other members (14) Influence concentrated among certain groups (14)	Creating power and participation equity (1, 4)
Context (total 50)		
Resource munificence (16/20)	Lack of (financial and human) resources (2, 4, 7, 11, 21, 24, 30) Financial barriers to sustainability beyond the project period (20, 29) Absence of strong funding partners (7) Sufficient resources available to individual organizations (10) Existing financial pressures (11)	High (financial and human) resource munificence (1, 2, 5, 23, 33) Having a greater number of revenue types (19) Central resource inventories (4) Receiving regular funding (28)
(Institutional) environment (10/14)	Regulatory or legislative barriers (5, 7, 24, 29) Different geographical boundaries (11) Different legislations and regulations for member organizations (22) Events occurring outside the project that have a direct impact on progress and/or success (29)	Understanding and making sense of the complex and changing health care environment (3, 20) Getting broad-based support from and participation within the community (2, 3) Urban locations (5) Recognition of specific local contexts and starting points (11) Aligning internal competencies with context (19)
Pre-existing relationships and attitudes toward collaboration (9/9)	Existing strained relationships (11)	Positive attitude toward or previous experience with collaboration (15, 28) Historical/previous relationships (1, 24) Informal collaboration/social interaction between members (23, 33) Perceived effectiveness to date (9) Mutual acquaintanceship (27)
Competition (3/3)	Competition from other entities that perform similar work as the network (2) Competitiveness among staff (7)	Finding a good balance between collaboration/competition (15)
System stability (2/2)	Increase in per capita income (5) Unexpected external influences such as national reforms (24)	
Mandate/ top-down/ bottom-up (1/1)	The free nature of cooperating in the network (21)	
Recognized interdependence (1/1)		A higher level of mutual benefit (6)
Structure (total 48)		
Network composition/ members (13/17)	Large number of member organizations (5, 12, 32) Difficulty recruiting the right members (21, 22, 29) Higher proportions of private sector, nonhealth organizations in partnership (5) Variation in organizational structures (29) The financing body and/or regulator of the network is also one of its members (32)	Member diversity (5, 6, 8, 25) Active soliciting of organizational member participation (1) Attracting and keeping people with the skills, talents, and connections needed (2) Focus on the recruitment of service providers instead of non-service providers (10) Careful selection of members but making sure the collaborative is not to small (33)
Governance (10/11)	Lack of a model for collaboration (22) Absence of champions (29) Combination of low levels of relational governance and trust (32)	Establishing the right governance structure (1, 7) Appointment of a lead or champion (11, 24) Social service agency as lead agency instead of medical lead agency (10) High level of formalization (22) Combining contractual, relational, and hierarchical governance mechanisms (32) Sponsorship that is aligned with the network’s values and aims (33)
Dynamic/ stability (5/6)	Turnover in leaders of the alliance or in member organizations (5, 18) Concurrent organizational change in members (24) Changes in partners/levels of involvement during the project (29)	Willingness to let structure follow strategy (structure seen as adaptable) (3) Changes in program director (18)
Interorganizational links (4/7)		Cliques (6, 23) High level of centrality (23, 25) High level of multiplexity (23) Referral heterophily and density provided conflicting results (25) Betweenness centralization, when one service tends to broker relationships with all other services (27)
Financing (3/7)	Creating a separate finance stream within existing pooled budget arrangements (11) Arrangements for sharing financial risk (11) Greater dependence on contracts relative to grants (19) Financial disincentives such as missing fee-for-service payment when attending network meetings (23)	Having a greater number of revenue types (19) Greater dependence on membership dues or contributions relative to grants (19) Implementing a membership dues structure (19)

#### Processes

Processes constitute the largest group of identified determinants (185 determinants in 18 themes). First, the problem, goal, and mission of the network are discussed most often in this group (19 articles, see Table 2). According to the reviewed studies, networks require a clear strategic direction (e.g., Alexander et al., 2010) and a shared, collectively agreed-upon problem, goal, and mission (e.g., D’Aunno et al., 2019; Metzger et al., 2005). Other studies show that a narrowly defined scope (Ling et al., 2012) and a well-defined target population (Conrad et al., 2003) facilitate network effectiveness. A network benefits from having a few programs and goals instead of having too many programs and goals and not being able to excel at them (Alexander et al., 2016). To reach a consensus on the network’s goal, it is beneficial to actively communicate with members and educate them on the network’s goals and activities and how these will affect them (Hearld, Alexander, Beich, et al., 2012) to ensure they are perceived as valuable by the members (Alexander et al., 2016; Ling et al., 2012).

Second, leadership is mentioned in 16 articles (see Table 2). Articles report that it is important to involve key leaders from a broad and representative group (Alexander et al., 2010) in the network who are aware of its initiatives (Alexander et al., 2016). Turnover of leaders in member organizations or the network’s director hampers network effectiveness (Bazzoli et al., 2003; Hearld et al., 2015), while changes in program managers are said to increase effectiveness (Hearld et al., 2015). In addition, leaders in networks often lack a set of skills that are deemed necessary to manage partner interactions (Sharek et al., 2007) and keep network members enthusiastic and focused on the network’s goals (Alexander et al., 2016; Sharek et al., 2007). Other key characteristics of network leaders include action-oriented (Bazzoli et al., 2003), empowering (Hearld & Alexander, 2014), ethical (Hasnain-Wynia et al., 2003), and supportive (Lemieux-Charles et al., 2005). Third, for planning and coordination mechanisms, articles report that networks require to adopt an appropriate time frame for their activities, make sure time frames are not too short to implement the initiatives (Ling et al., 2012), and match the time frames of network members (Sharek et al., 2007). Being task-oriented (Alexander et al., 2003) and clearly defining roles and responsibilities, especially between network members and staff (Alexander et al., 2010; Bazzoli et al., 2003), facilitate network effectiveness. Networks also benefit from planning specific improvement routes (Nikbakht-Van de Sande et al., 2005) integrating individual components (Conrad et al., 2003), aided by standards of practices (Bainbridge et al., 2011). In addition, involving stakeholders in network activities as early as possible facilitates network effectiveness (van Eyk & Baum, 2002).

Fourth, decision-making processes are mentioned in 12 articles (see Table 2). While articles agree that it is beneficial for decision-making processes to be collaborative (D’Aunno et al., 2017) and inclusive (Hearld et al., 2013), giving every member of the network a voice in decision-making (Hearld & Alexander, 2014), studies also show that decision-making processes are contingent and thus need to be congruent with the type of decision that is being made (Hearld et al., 2013). A voting system (Bainbridge et al., 2011) or formal framework (Hearld et al., 2013) may facilitate decision-making processes. Finally, individuals at the operational level (i.e., care professionals such as doctors and nurses) seem to play an important role in network effectiveness (10 articles). Articles report barriers to network effectiveness if care professionals were reluctant to engage in network activities (Ling et al., 2012) or when their interest in network activities waned (Sharek et al., 2007). Therefore, it is beneficial to involve doctors and other professionals directly affected by changes due to network activities in the network (Hermans et al., 2019; Lemieux-Charles et al., 2005; Wilson et al., 2003). Other articles stress that creating high levels of awareness of network activities throughout all hierarchical levels in member organizations (Hearld, Alexander, & Mittler, 2012) fostering shared beliefs on the (personal) benefits from changing work patterns and integrated working (Harlock et al., 2020; Ling et al., 2012) facilitate network effectiveness.

#### Context

In the group context (50 determinants in 7 themes), the theme influencing network effectiveness mentioned most often is resource munificence (16 articles, see Table 2). Articles show that networks experience difficulties to collect enough resources to be able to achieve their goals or to create sustainability beyond the project period and implement the initiative into routine practice (e.g., Hearld et al., 2019; Sharek et al., 2007). These resources often involve financial resources (e.g., Alexander et al., 2016; Hermans et al., 2019) but may also include human resources (e.g., Harlock et al., 2020; Lemieux-Charles et al., 2005). Articles find that networks can increase their effectiveness when they receive regular funding (Nikbakht-Van de Sande et al., 2005), have strong funding partners (Conrad et al., 2003), establish central resource inventories (Bainbridge et al., 2011), and focus on having a greater number of revenue types (Hearld et al., 2018).

Second, the (institutional) environment of networks is mentioned in ten articles (see Table 2). Networks experienced legislative or regulatory barriers such as delayed state approval for the network’s health plan (Conrad et al., 2003) or different legislations and regulations across sectors, inhibiting collaboration, data sharing, and integrated initiatives (Kristensen et al., 2019; Sharek et al., 2007). In addition, members in networks may cover different geographical areas and, as such, serve different populations, creating problems in implementing initiatives (Harlock et al., 2020). Therefore, articles note that it is important to understand the local contexts of member organizations and their starting points, such as the local workforce and provider markets (Harlock et al., 2020), as well as the broader, complex health care environment (Alexander et al., 2003), such as changes in regulations that may lead to shifting goals and values of member organizations (Alexander et al., 2016).

Finally, preexisting relations and attitudes toward collaboration are mentioned in nine articles (see Table 2). Harlock et al. (2020) report that existing relationships that were strained created tensions within the network, inhibiting network effectiveness. Nevertheless, an extensive history of collaboration and existing good relationships (Hearld, Alexander, Beich, et al., 2012; Ling et al., 2012), especially informal collaboration and social interaction within and outside the formal network boundaries (e.g., Lemieux-Charles et al., 2005; Wilson et al., 2003), are also favorable for network effectiveness. A positive attitude toward networks and collaboration in general (Nikbakht-Van de Sande et al., 2005) and perceived effectiveness of the current network to date (D’Aunno et al., 2019) were also identified as facilitators of network effectiveness.

#### Structure

The theme influencing network effectiveness most often mentioned in the group “structure” (48 determinants in 5 themes) is the composition of the network (13 articles, see Table 2). Articles describe that recruiting organizational members to participate in the network is difficult (Hermans et al., 2019; Kristensen et al., 2019), yet active soliciting is necessary to attract the skills, talents, or political connections needed for the network to be effective (Alexander et al., 2010, 2016). While member diversity is often mentioned as a facilitating determinant (e.g., D’Aunno et al., 2017), especially among members who offer differentiated services to similar client groups (Bunger & Gillespie, 2014), articles also found that a large number of members in the network is an inhibiting determinant (e.g., Bazzoli et al., 2003; Hasnain-Wynia et al., 2003). Second, Alexander et al. (2010) delineate that whether to adopt a completely new governance structure (10 articles, see Table 2) or an already existing one both has its advantages and disadvantages. However, it may have implications for how the network will be perceived by external and internal stakeholders, and consequently, how well the network will be able to recruit members and generate resources. The presence of champions, (staff) members that articulate the network’s vision to the team level and coordinate and manage the implementation of initiatives, is identified by articles as a facilitating determinant (Harlock et al., 2020; Ling et al., 2012; Sharek et al., 2007).

### Type of Effectiveness

Table 3 presents a summary of the measure of effectiveness used in the included studies, the data collection approach, and the type of respondents. Most studies (*n* = 21) measured effectiveness in relation to a process measure (see Table 3), such as the degree of consensus among network members on a problem or course of action (e.g., Hearld et al., 2013), the quality of collaboration in the network (e.g., Nicaise et al., 2021), or sustaining member participation (Hearld & Alexander, 2020). Most of these studies used surveys and/or interviews with network participants as their primary source of data. Two studies focusing on process outcomes also included community representatives (Hasnain-Wynia et al., 2003) or consumers (Hearld & Alexander, 2020) as respondents. One study included researchers as respondents (Sharek et al., 2007). In seven studies, effectiveness was measured as the degree to which the network attained its goals of increasing the quality and/or decreasing the cost of care delivery, as per our inclusion criteria (see Table 3). One of these studies used qualitative evaluation reports, one reported patient-level outcomes (Lorant et al., 2017), and five studies reported only perceptions of network participants on the degree of goal attainment (see Table 3). Network sustainability was reported as a measure of effectiveness in six articles. One article reported the amount of change that occurred in member organizations (Hearld, Alexander, & Mittler, 2012), and two articles did not specify the measure of effectiveness (Hermans et al., 2019; Wilson et al., 2003).

**Table 3.** Classification of the Measure of Effectiveness, Data Collection, and Respondents.

Type	Article	Measure of effectiveness^a^	Data collection	Respondents
Process measure (21)	2	Sustainability of the network and how well the networks were positioned to accomplish their goals	Interviews and survey	Network participants
	3	Sustainability of the network, developmental progress in realizing the objectives of the partnership, the capacity to collaborate, and a stable financial base	Interviews	Network participants
	4	The extend of interorganizational collaboration and network sustainability	Interviews and survey	Network participants
	5	Successful implementation of initiatives and completion of intermediate action steps	Interviews and survey	Network participants
	8	Cost and benefits for members of their participation the network over time	Survey	Network participants
	10	Retaining individual agencies as part of the network	Interviews and survey	Network participants
	11	Experiences of implementation of the initiative and perceived impacts	Interviews and group interviews	Network participants
	12	Participants’ perceptions of their own network’s effectiveness	Survey	Network participants (including community representatives)
	13	The network’s ability to galvanize participants to take action and institutionalize the vision, goals, and programs	Survey	Network participants
	14	Sustaining member participation	Survey	Network participants (including consumers)
	15	A shared understanding about the network’s vision, strategic goals, and sense of commitment, collaboration, and cooperation among stakeholders	Interviews and survey	Network participants
	16	Level of consensus among members	Survey	Network participants
	18	The costs and benefits members derive from participating in the network	Survey and interviews	Network participants
	20	Dissemination of innovative practices to improve the health and quality of care in their local communities	Survey and interviews	Network participants; documents included financial worksheets
	23	Agency-level and network-level, administrative and service-delivery effectiveness in each network	Survey	Network participants
	26	Benefits and costs of participation in the network	Survey	Network participants
	27	The quality of collaboration	Survey	Network participants
	28	More effective collaboration and perceived enhanced quality of care	Interviews and survey	Network participants
	29	Network-based implementation results in overcoming the identified barrier	Survey	Principal investigators
	30	Degree of and perception toward collaboration	Survey and interviews	Network participants
	31	Cooperation in the network: that participants have made and kept to agreements	Interviews and documents	Network participants; documents included internal policy/ evaluation documents
Goal attainment (i.e. value of care delivery) (7)	6	Perceptions whether the network benefited efficiency of services, client access to services, and quality of care	Survey and secondary data	Network participants; online financial and organizational data
	7	Measures of process changes measured along four components (community accountability, community health focus, managing under-limited resources, and seamless continuum of care) and four intermediate outcomes (access, cost, quality of service delivery, and health outcomes)	Interviews, survey, and documents	Network participants; documents included program reports
	22	Continuity of care across sectors	Documents	Qualitative network evaluation reports were used only to extract determinants of effectiveness
	24	Effectively delivering integrated services as perceived by staff involved in pilots	Interviews	Network participants
	25	Experienced continuity of care and social integration at the patient level	Two surveys	One with network participants; one with patients
	28	More effective collaboration and perceived enhanced quality of care	Interviews and survey	Network participants
	32	Perceptions of whether the network was reaching its goals	Interviews and survey	Network participants
Network sustain-ability (6)	1	Capacity building to sustain activity and participation	Interviews	Network participants
	2	Sustainability of the network and how well the networks were positioned to accomplish their goals	Interviews and survey	Network participants
	3	Sustainability of the network	Interviews	Network participants
	4	The extent of interorganizational collaboration and network sustainability	Interviews and survey	Network participants
	9	Sustainability of the network	Survey	Network participants
	19	Sustainability of the network	Survey, interviews, and documents	Network participants; documents included financial worksheets
Other (3)	17	The amount of change that occurred in member organizations as a result of the alliance and its activities	Survey	Network participants
	21	Not specified	Survey and focus groups	Network participants
	33	Not specified	Interviews	Network participants

a Measure of effectiveness of four articles (2, 3, 4, and 28) was classified into two categories.

## Discussion

The purpose of this review was to understand which determinants influence various types of the effectiveness of purpose-oriented networks in health care. Based on our initial search, backward snowballing procedure, and expert consultation, 33 articles were included in our analysis, most of which utilize small samples at the network level. Our results show 283 determinants of effective health care networks, clustered into 30 themes. The group processes encompass almost two thirds of the determinants identified, while the context and structure are less frequently studied. In the group processes, the involvement of care professionals from the operational level plays a prominent role in purpose-oriented networks in health care. In addition, our results show that most empirical work focuses on the determinants of the collaborative process within networks, whereas the determinants of networks’ (perceived) goal attainment are less frequently described. In what follows, we reflect on these results.

First, our findings show that literature has mainly focused on processual determinants of network effectiveness at the expense of contextual and structural determinants. In doing so, the network literature seems to underemphasize the heavily institutionalized nature of the health care context (Reay et al., 2016), which is considered an important feature of the sector. Similarly, research has revealed the necessity of adequate organizational structures for good health care delivery (Cowen et al., 2008). Given the complexity of networks as organizational forms (Nowell & Kenis, 2019), increased focus on structural and contextual determinants of their effectiveness could advance our ability to make them effective.

Second, the 283 determinants across 30 themes we identified are predominantly described in reductionist, “net-effect,” relationships to network effectiveness. That is, the role of a specific determinant is often singled out, without considering it in combination with other determinants. While this is the case in network research more broadly (Smith, 2020), researchers of networks have been arguing that the determinants of network effectiveness follow complex configurations and mechanisms (Provan & Milward, 1995; Raab et al., 2013). It is thus imperative that we start to understand these using configurational methods (Smith, 2020). One methodological approach to studying causally complex phenomena and identifying whether and how combinations of determinants produce certain outcomes is Qualitative Comparative Analysis (QCA; Misangyi et al., 2017; Ragin, 1987). The QCA method furthermore allows for valid interpretation of results drawn from studies with small sample sizes (Misangyi et al., 2017). Our results show that most studies of health care networks do not exceed a sample size of 25 at the network level. This might be attributed to the fact that gaining access to such networks and the multiple organizations within these networks is not easy (Hearld & Westra, 2023) and studying networks is a labor-intensive task because of the multilevel nature even within one single case. The QCA approach may thus prove especially useful for the study of networks in health care.

Third, our findings emphasize the importance and difficulty of involving health care professionals (i.e., the operational level) for the effectiveness of health care networks. These findings are in line with previous research, which has shown that collaboration in social networks occurs in and across multiple levels (Lomi et al., 2015) and that involving different organizational levels is beneficial for decision-making within individual organizations (Harrington & Ottenbacher, 2009). Nevertheless, involving the operational level did not appear as a determinant in any of the previous overviews of network effectiveness outside the specific health care context (Ansell & Gash, 2007; Bryson et al., 2015; Parent & Harvey, 2009; Planko et al., 2017; Popp et al., 2014; Smith, 2020; Turrini et al., 2010). The importance of the operational level in health care networks could be explained by the high level of autonomy of health care professionals and, consequently, the resistance often encountered when changes occur in the roles of these professionals (Mintzberg, 1993; Reay et al., 2016), which are typically initiated by decisions taken in the network (Hearld & Westra, 2023). However, little is known about the specific multilevel mechanisms through which professionals contribute specifically to network effectiveness or the dynamics between professionals and organizational representatives at the strategic and tactical levels within these networks (Hearld & Westra, 2023). These insights could contribute to our understanding of networks in health care in particular and professional service industries more broadly. In the meanwhile, health care executives and managers would do well to include key professionals in the organization and operationalization of network activities to increase their effectiveness.

Finally, our finding that most empirical work measures effectiveness as a process outcome rather than a goal attainment measure shows that literature has mainly identified determinants of “good” collaboration at the level of the network, whereas our understanding of the determinants of outcomes at the level of the client of community, such as improving the value (i.e., increasing quality and/or decreasing cost) of care delivery, remains limited. Literature thus focuses on network effectiveness at the level of the network itself but overlooks effectiveness at levels such as that of the community and individual clients (Provan & Milward, 2001). This is particularly problematic as theory shows that network effectiveness at one level (e.g., the network) may be generated at the expense of effectiveness at other levels (e.g., the patient; Smith, 2020). While relational coordination predicts improved (hard) outcomes (Gittell, 2006), these outcomes are currently outweighed by process measures. The studies that did define effectiveness as goal attainment assessed effectiveness based on the perception of network members. Although perceived effectiveness is important to retain the commitment of its members (Turrini et al., 2010) and the sustainability of the network (Ahrne & Brunsson, 2005), the positive perception toward networks may enhance the institutionalization of networks as an effective way to address these wicked problems (Selznick, 1996), without understanding the most important determinants to do so effectively. Measuring networks’ goal attainment in terms of lowered costs or improved quality may pose challenges for researchers due to the fragmented nature of health systems and its financial arrangements (Stange, 2009) as well as the resistance of organizations to share this data (e.g., insurers). However, research on system-oriented networks (Brewster et al., 2019) and Accountable Care Organizations (McWilliams et al., 2016) shows that it is possible to acquire these kinds of data. To ensure effectiveness at the network level is not created at the expense of effectiveness at the client or community level, such as cost or quality of care, future research also needs to assess the determinants of networks’ goal attainment using objective data in addition to process and perceived measures.

### Limitations

This study’s limitations mainly stem from the fragmented nature of the literature and the multiple labels and definitions used to describe networks (Lemaire et al., 2019). Therefore, we had to rely solely on MeSH-terms or Exact Major Subject headings to identify studies related to purpose-oriented networks. Our search could therefore overlook studies that did not use the appropriate MeSH terms or Exact Major Subject Headings. However, the use of free-text terms would yield a near unwieldable number of studies. In addition, we utilized a rather stringent set of inclusion criteria, only including studies assessing determinants influencing the effectiveness of purpose-oriented networks aiming to improve the value of health care delivery. While many studies, from our perspective, failed to meet these criteria, such as structural-oriented networks (e.g., professional networks), system-oriented networks (e.g., the work of Provan & Milward, 1995), or networks targeting social determinants of health, they were necessary to ensure the included articles studied the same type of network (i.e., purpose-oriented networks) in similar contexts (i.e., health care delivery). While different types of networks might learn from each other in some respects, the fundamental differences between them also ask for separate analyses (Nowell & Milward, 2022). We should note, however, that this is one of the first studies applying this taxonomy to empirical phenomena. Nowell and Milward (2022) also recognized that some networks might be classified or might evolve into multiple types of networks, such as a structural-oriented network evolving into a purpose-oriented network. Consequently, different research might have different interpretations on the three classes of networks applied in this study, leading to other choices when in- and excluding articles. There is a need for a common language to be able to draw systematic meta-analyses on network studies.

## Conclusion

Deliberate networks between multiple organizations are seen as (part of) the solution to the wicked problems in health care. Empirical literature describes an abundance of determinants that influence the effectiveness of these networks and involving health care professionals in the network activities and leadership appears to be especially salient in professional service networks. However, most of the determinants of effective health care networks are processual in nature, are described though a reductionist perspective, and are related to process outcomes rather than goal attainment measures of effectiveness. Consequently, our understanding of network effectiveness remains limited to perceptions on how to collaborate instead of how networks can actually achieve their goals of improving value in health care delivery. Future research can address this gap using configurational approaches and measures of networks’ goal attainment.
